# Planning evaluation of a novel volume-based algorithm for personalized optimization of lung dose in VMAT for esophageal cancer

**DOI:** 10.1038/s41598-021-04571-3

**Published:** 2022-02-15

**Authors:** Chen-Xiong Hsu, Kuan-Heng Lin, Shan-Ying Wang, Wei-Ta Tsai, Chiu-Han Chang, Hui-Ju Tien, Pei-Wei Shueng, Tung-Hsin Wu, Greta S. P. Mok

**Affiliations:** 1 Department of Biomedical Imaging and Radiological Sciences, National Yang Ming Chiao Tung University, Taipei, Taiwan; 2 Division of Radiation Oncology, Far Eastern Memorial Hospital, New Taipei City, Taiwan; 3 Industrial Ph.D. Program of Biomedical Science and Engineering, National Yang Ming Chiao Tung University, Taipei, Taiwan; 4 Department of Nuclear Medicine, Far Eastern Memorial Hospital, New Taipei City, Taiwan; 5 Faculty of Medicine, School of Medicine, National Yang Ming Chiao Tung University, Taipei, Taiwan; 6 Biomedical Imaging Laboratory, Department of Electrical and Computer Engineering, Faculty of Science and Technology, University of Macau, Macau, SAR China

**Keywords:** Oesophageal cancer, Radiotherapy

## Abstract

Radiotherapy treatment planning (RTP) is time-consuming and labor-intensive since medical physicists must devise treatment plans carefully to reduce damage to tissues and organs for patients. Previously, we proposed the volume-based algorithm (VBA) method, providing optimal partial arcs (OPA) angle to achieve the low-dose volume of lungs in dynamic arc radiotherapy. This study aimed to implement the VBA for esophageal cancer (EC) patients and compare the lung dose and delivery time between full arcs (FA) without using VBA and OPA angle using VBA in volumetric modulated arc therapy (VMAT) plans. We retrospectively included 30 patients diagnosed with EC. RTP of each patient was replanned to 4 VMAT plans, including FA plans without (FA-C) and with (FA + C) dose constraints of OARs and OPA plans without (OPA-C) and with (OPA + C) dose constraints of OARs. The prescribed dose was 45 Gy. The OARs included the lungs, heart, and spinal cord. The dose distribution, dose-volume histogram, monitor units (MUs), delivery time, and gamma passing rates were analyzed. The results showed that the lung V_5_ and V_10_ in OPA + C plans were significantly lower than in FA + C plans (*p* < 0.05). No significant differences were noted in planning target volume (PTV) coverage, lung V_15_, lung V_20_, mean lung dose, heart V_30_, heart V_40_, mean heart dose, and maximal spinal cord dose between FA + C and OPA + C plans. The delivery time was significantly longer in FA + C plans than in OPA + C plans (237 vs. 192 s, *p* < 0.05). There were no significant differences between FA + C and OPA + C plans in gamma passing rates. We successfully applied the OPA angle based on the VBA to clinical EC patients and simplified the arc angle selection in RTP. The VBA could provide a personalized OPA angle for each patient and effectively reduce lung V_5_, V_10,_ and delivery time in VMAT.

## Introduction

With the rapid development of dynamic arc radiotherapy, volumetric modulated arc therapy (VMAT) and tomotherapy could have better tumor coverage of the treatment plans for esophageal cancer (EC). However, increased low-dose exposure to the lungs is observed due to the continuous rotation of the gantry^1–4^. Radiation pneumonitis (RP) is one of the severe complications after radiotherapy for EC patients. Meanwhile, the relative lung volume receiving more than 5 Gy (V_5_) and 20 Gy (V_20_) and mean lung dose (MLD) are important dosimetric factors for RP^5–8^.

Many methods for reducing the lung dose have been reported in dynamic arc radiotherapy^9, 10^. However, the selection of gantry arc angle and dose constraints are the key factors in radiotherapy treatment planning (RTP). To reduce the radiation dose to the lungs, the medical physicists usually manually adjust the optimization parameters, which is complex and time-consuming in inverse treatment planning. It took an average of 3.8 h to complete the EC treatment plan manually^11^. Several automatic planning techniques were developed in RTP to reduce the planning time to 62–155 min^12, 13^. A high-quality treatment plan could provide high tumor coverage, low normal tissue dose, and a shorter delivery time in VMAT, e.g., an average of 6.1–6.6 min to deliver the total monitor unit (MUs)^10, 14^.

Previously, we proposed the volume-based algorithm (VBA) method to quickly calculate the optimal partial arcs (OPA) angle corresponding to the lung V_5_ by defining the length and width of the planning target volume (PTV)^15^. We demonstrated that VBA could improve the efficiency of VMAT planning to reduce the lung V_5_ within 5 min in a phantom study. The purpose of this study was to implement the OPA angle based on the VBA for clinical EC patients and to compare the lung dose and delivery time between full arcs (FA) without using VBA and OPA angle using VBA in VMAT plans.

## Materials and methods

### Patient population and study design

**Figure 1:**
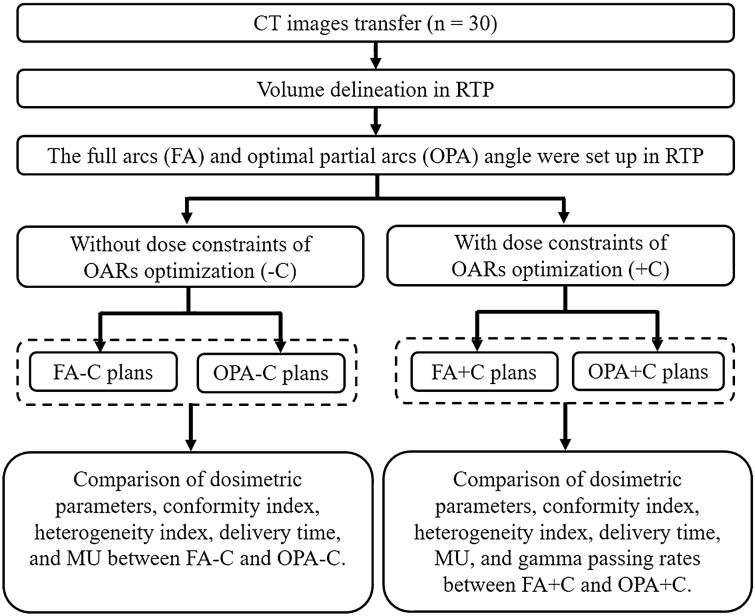
Flowchart of study design.

Thirty EC patients were retrospectively included in this study. Each patient was replanned retrospectively to 4 VMAT plans. Figure 1 shows the flowchart of the study design. First, the computed tomography (CT) images were transferred to RTP. The PTV and organs at risk (OARs) were delineated in RTP. The FA angle without using VBA was set for each patient. Each personalized OPA angle could be calculated by using VBA. FA plans without (FA-C) and with (FA + C) dose constraints of OARs and OPA plans without (OPA-C) and with (OPA + C) dose constraints of OARs were generated. The dosimetric parameters, conformity index (CI), heterogeneity index (HI), delivery time, MUs, and gamma passing rate were assessed in 4 VMAT plans for each patient. The results of the FA-C and OPA-C plans are shown in the supplementary.

### Volume delineation

The CT images were transferred to the Pinnacle treatment planning system (TPS) (version 9.8; Philips Medical Systems North America, Andover, MA, USA) to delineate the targets and OARs. The targets delineated included the gross tumor volume (GTV), clinical target volume (CTV), and planning target volume (PTV) by radiation oncologists. The GTV covered the visible primary tumor and gross lymph nodes on the CT images. The CTV was designed to cover a region with subclinical disease from GTV by expanding 1 cm superiorly and inferiorly and 0.5 cm laterally on both left and right sides, anteriorly and posteriorly^16^. The organ movements caused by breathing, swallowing, and position uncertainty in each therapy were considered when defining the PTV. In accordance with clinical experience, the PTV was defined by expanding the CTV on three dimensions by 0.8 cm^17^. The OARs delineated included the lungs, heart, and spinal cord by the medical physicist.

### The optimal partial arcs angle generation in VBA

**Figure 2:**
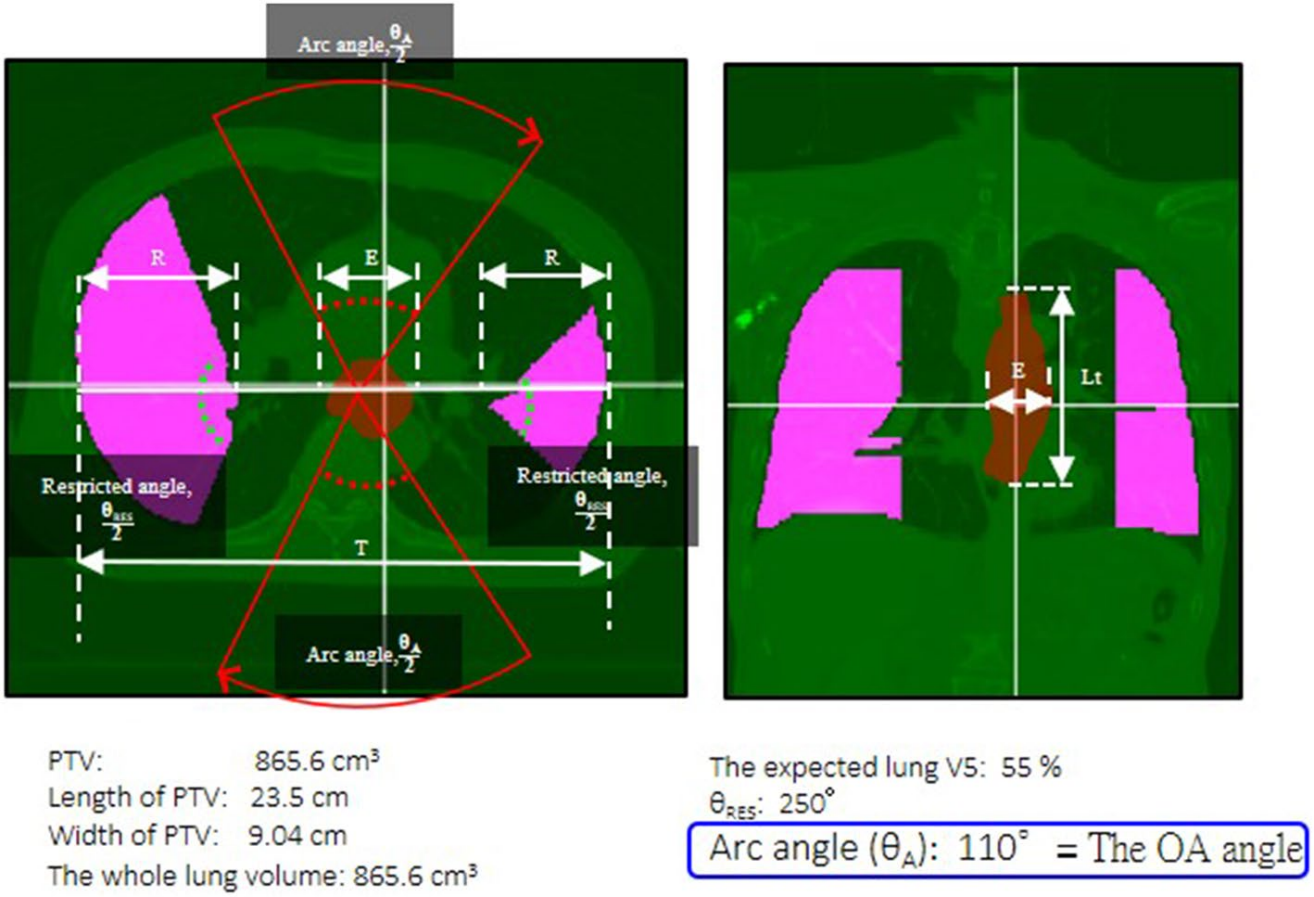
Sample patient for calculating the OPA angle in VBA. The axial and coronal views are shown on the VBA interface. The PTV (red area), length and width of PTV, lung volume, and the expected lung V_5_ were shown as input. When the lung V_5_ was less than 55%, the θ_A_ was 110° (solid red line), indicating an OPA angle of 110° for this patient. The pink area is restricted volume.

Before the treatment arc angle selection, the personalized OPA angle could be calculated using VBA for each patient according to Eqs. (1)–(2)^15^. The width of the PTV (E) was measured on the axial plane, while the axial length of the PTV (Lt) was measured vertically on the coronal image of the centroid of the PTV. The transverse diameter of the thorax (T), the radius of one side of the restricted volume (R), E, Lt, the whole lung volume (V_W_), the total volume out of the field (V_OW_), and the expected lung V_5_ were input to the VBA to obtain the personalized OPA angle. When the lung V_5_ was less than 55%, the θ_A_ would be chosen as the OPA angle (Fig. 2). The OPA angle could then be applied in the RTP. Each OPA angle of different patients would be calculated before the RTP optimization.

1 $$\text{R} =\frac{ {\text{T} - {\text{E}}} - 4}{2}$$

where T is the transverse diameter of the thorax, R is the radius of one side of the restricted volume, and E is the width of the PTV.

The R, Lt, V_W_, and V_OW_ are then input into Eq. (2) to obtain the θ_A_, which was the personalized OPA angle:

2 $$\uppi {\text{R}}^{2}\frac{{360-\uptheta }_{\mathrm{A}}}{360^\circ }\left(\mathrm{Lt}+4\right)+{\mathrm{V}}_{\mathrm{OW}}={\mathrm{V}}_{\mathrm{W}}\times 0.45$$

where Lt is the length of the PTV, V_W_ is the whole lung volume, and V_OW_ is the total volume out of the field.

### Radiation treatment planning optimization

The FA and OPA plans were performed using the Pinnacle TPS with the 6-MV photon beam. The FA plans used two full arcs. One arc was set up in a clockwise (CW) direction from 180° to 179°; conversely, the second arc was performed in a counterclockwise (CCW) direction from 179° to 180°, and the collimator was rotated 5° extra to reduce the overlapping tongue and groove effects. According to tumor size, the OPA angle was calculated in the VBA.

Due to the limitation that the gantry of an Elekta Versa HD linear accelerator cannot pass from 180° to − 180°, the OPA angle was divided into six partial arcs in OPA plans. Three CW partial arcs were from 180 to $\left(180 + \frac{{\uptheta }_{\mathrm{A}}}{4}\right), \left(-\frac{{\uptheta }_{\mathrm{A}}}{4}\right)$ to $\frac{{\uptheta }_{\mathrm{A}}}{4}$ , and (180 − $\frac{{\uptheta }_{\mathrm{A}}}{4})$ to 179; conversely, Three CCW partial arcs were from 179 to (180 − $\frac{{\uptheta }_{\mathrm{A}}}{4})$, $\frac{{\uptheta }_{\mathrm{A}}}{4}$ to $(-\frac{{\uptheta }_{\mathrm{A}}}{4})$, and (180 + $\frac{{\uptheta }_{\mathrm{A}}}{4})$ to180. The VMAT fields were inversely planned and optimized using SmartArc module optimization in Pinnacle TPS in FA and OPA plans with the following parameters: stopping tolerance of 10-7, constraint leaf motion of 0.46 cm/°. The maximum delivery time was set to 120 and 40 s per arc for FA and OPA plans with a gantry angle spacing of 4°. The dose distributions were calculated with the adaptive convolution method.

A prescribed dose of 45 Gy in 25 fractions (1.8 Gy per fraction) was defined. The goal was to cover 95% of each PTV with the prescribed dose (D_95_). The dose constraints for OARs were defined as follows based on the RTOG 1010 and the related studies^18, 19^: the maximum dose was < 45 Gy for the spinal cord; the mean heart dose (MHD) was < 34 Gy and the V_40_ of heart was < 50%; the MLD for the whole, right, and left lung must be < 20 Gy; the whole, right, and left lung V_20_, V_15_, V_10_, and V_5_ were ≤ 20%, ≤ 30%, ≤ 50%, and ≤ 55%, respectively. The dose constraints were adjusted to cover adequate and homogeneous target volume during the dose optimization process while minimizing the dose in the heart, spinal cord, and lungs.

### Plan evaluation

The PTV coverage was evaluated using the CI and the HI.

The HI^20^ was defined as the following equation:

3 $$\mathrm{HI}=\frac{{\mathrm{D}}_{5\mathrm{\%}}}{{\mathrm{D}}_{95\mathrm{\%}}}$$

where D_5%_ is the minimum dose delivered to the 5% of the PTV, and D_95%_ is the minimum dose in 95% of the target volume. The HI closer to 1 indicates better dose homogeneity. An index between 1.00 and 1.40 is acceptable^21^.

The CI^22^ was defined as Eq. (4):

4 $$\mathrm{CI}=\frac{{\mathrm{V}}_{\mathrm{RI}}}{\mathrm{TV}}$$

where V_RI_ is reference isodose volume and TV is target volume. The CI closer to 1 indicates good target conformity and coverage. An index is acceptable between 0.9 and 2. An index between 2 and 2.5, or 0.9 and 1, is tolerable^22^.

The information provided by the dose-volume histogram (DVH) in the RTP of the following parameters were recorded, i.e., MLD, lung V_5_, lung V_10_, lung V_15_, lung V_20_, MHD, heart V_30_, heart V_40_, and the spinal cord maximum dose.

The FA + C and OPA + C plans were checked for the deliverability of the plan. Sixty VMAT plans underwent dose verification on the treatment machine using the Octavius phantom (Octavius II, PTW Freiburg GmbH, Freiburg, Germany). The plans were assessed based on the gamma criteria of 3%/3 mm with a clinical passing threshold of 95% of points^23^.

### Statistical analysis

The SPSS software package (version 24.0; IBM Corporation., Armonk, NY, USA) was used for statistical analysis. Mann–Whitney test was used to compare the dosimetric parameters, CI, HI, delivery time, and MUs differences between FA and OPA plans. A *p* < 0.05 was considered statistically significant.

### Ethics approval and consent to participate

All experimental procedures were approved by the Research Ethics Review Committee of Far Eastern Memorial Hospital (No. 108069-E). The Research Ethics Review Committee of Far Eastern Memorial Hospital waived the need for informed consent. All research was performed in accordance with relevant guidelines and regulations.

## Results

### Patient characteristics

**Figure 3:**
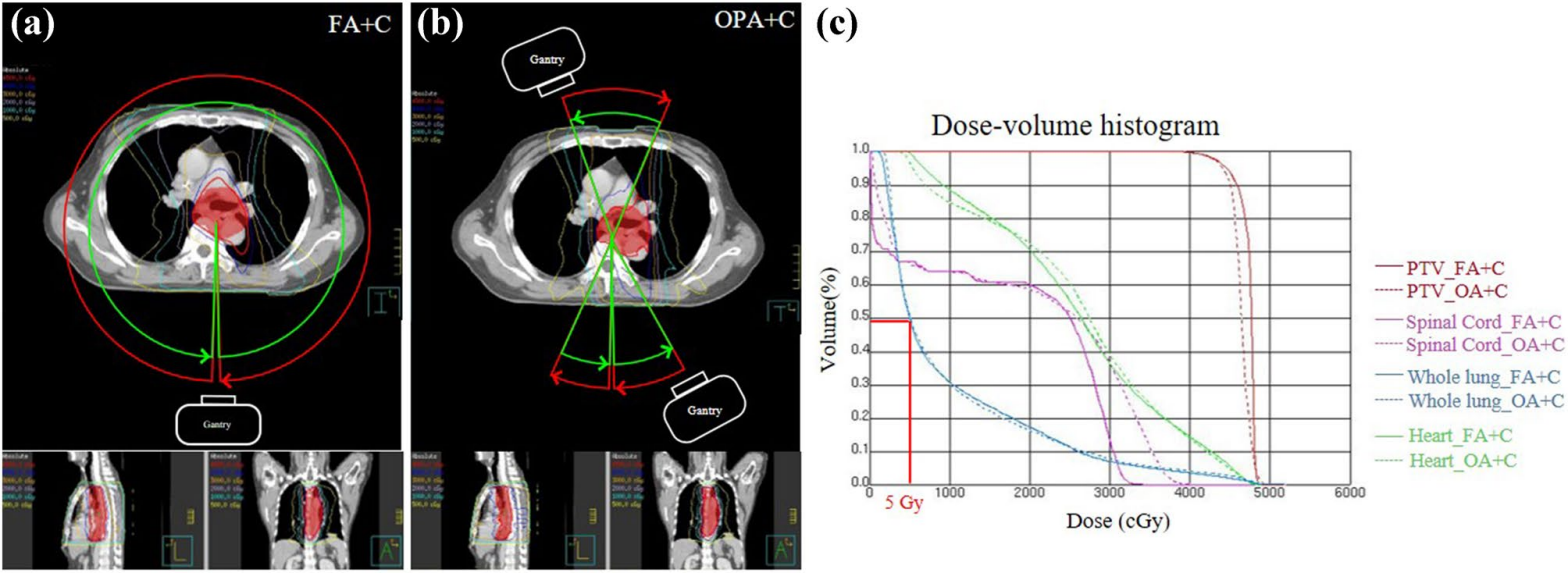
The isodose curves and DVH of VMAT plans for a patient. For this patient, the OPA angle was 110°. (**a**) Isodose curves of FA + C plan. (**b**) Isodose curves of OPA + C plan. (**c**) DVH for FA + C and OPA + C plan. The gantry arc angles were shown in (**a**) and (**b**). The red arcs were counterclockwise (CCW) direction. The green arcs were clockwise (CW) direction. The FA plans used two full arcs. The OPA plans used six partial arcs. The red line means the relative lung volume receiving more than 5 Gy (V_5_) in DVH of (**c**).

**Figure 4:**
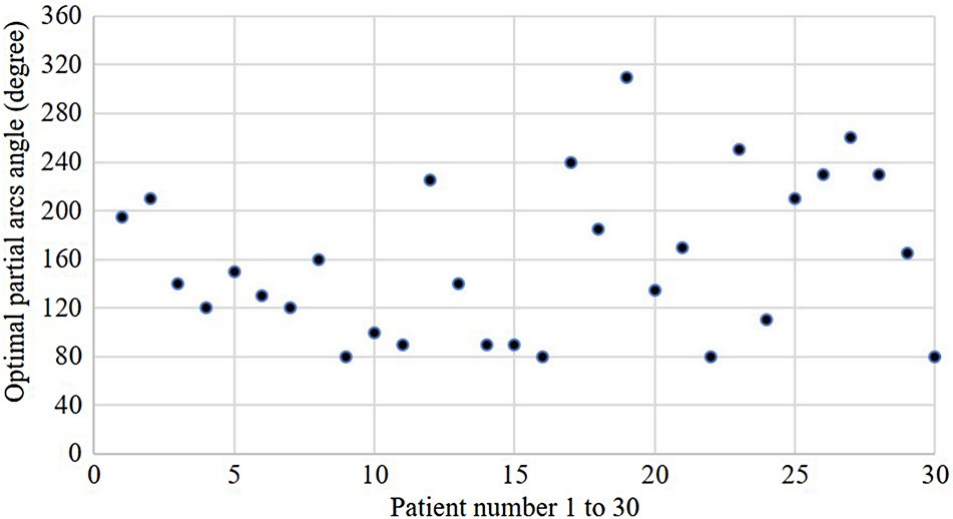
The range of OPA angles was from 80° to 310° for 30 patients. The OPA angle could be calculated using VBA for each patient.

Table 1 shows the detailed patient characteristics in 30 patients. Figure 3 displays the isodose curves and DVH of FA + C and OPA + C plans for a sample EC patient. The results showed that the PVT could achieve good target coverage and the OARs could also decline to the acceptable dose. The OPA angle for each patient is presented in Fig. 4. The range of the OPA angles was from 80° to 310°.

**Table 1:** Characteristics of EC patients in this study.

Characteristics		n = 30
Sex	Male	20
	Female	10
Age (years)	Median	56
	Range	43–78
Length of tumor (cm)	Median	18.65
	Range	6.30–25.20
PTV (cm^3^)	Median	769.80
	Range	281.53–1234.78
Location of tumor	Upper	3
	Middle	20
	Lower	7
T	1	15
	2	9
	3	6
N	0	10
	+	20
M	0	29
	1	1
AJCC stage	I	14
	II	8
	III	7
	IV	1

*PTV* planning target volume.

### PTV coverage and OAR sparing

Comparisons of the PTV coverage and OAR sparing between FA + C and OPA + C plans are shown in Table 2. The whole lung V_5_ and V_10_ in OPA + C plans were significantly lower than in FA + C plans (*p* < 0.05). The median of the lung V5 between FA + C and OPA + C plans were 48.5% (range 23.0–53.3%) and 44.5% (range 21.1–53.3%). No significant differences were noted in PTV coverage, MHD, heart V_30_, heart V_40_, and the spinal cord maximum dose between FA + C and OPA + C plans (Table 2).

**Table 2:** Comparison of dosimetric factors between FA + C and OPA + C plans.

Parameter		FA + C	OPA + C	*p*-value
PTV	D_5_ (Gy)	46.88 ± 6.99	47.53 ± 7.49	0.539
	D_95_ (Gy)	43.31 ± 6.99	43.29 ± 7.09	0.976
	HI	1.08 ± 0.03	1.10 ± 0.04	0.159
	CI	1.15 ± 0.18	1.18 ± 0.16	0.375
Whole lung	Mean dose (Gy)	10.37 ± 1.67	9.87 ± 1.82	0.252
	V_20_ (%)	18.11 ± 4.09	17.14 ± 4.03	0.414
	V_15_ (%)	23.75 ± 4.51	21.77 ± 4.57	0.094
	V_10_ (%)	30.89 ± 5.07	28.05 ± 5.59	0.041*
	V_5_ (%)	48.55 ± 6.82	43.38 ± 8.22	0.005*
Right lung	Mean dose (Gy)	9.81 ± 2.04	9.22 ± 2.16	0.237
	V_20_ (%)	16.75 ± 5.08	15.79 ± 5.02	0.454
	V_15_ (%)	21.79 ± 5.70	20.00 ± 5.62	0.219
	V_10_ (%)	29.25 ± 6.70	26.07 ± 6.72	0.041*
	V_5_ (%)	45.71 ± 8.81	40.79 ± 9.09	0.015*
Left lung	Mean dose (Gy)	10.95 ± 2.03	10.62 ± 2.21	0.706
	V_20_ (%)	19.25 ± 5.51	18.82 ± 5.04	0.689
	V_15_ (%)	24.93 ± 5.52	23.75 ± 6.02	0.553
	V_10_ (%)	32.79 ± 6.13	30.18 ± 7.00	0.149
	V_5_ (%)	50.46 ± 7.16	46.11 ± 9.77	0.033*
Heart	Mean dose (Gy)	20.76 ± 8.25	21.37 ± 8.30	0.813
	V_40_ (%)	12.54 ± 10.30	13.64 ± 10.45	0.728
	V_30_ (%)	29.21 ± 17.63	30.96 ± 17.22	0.695
Spinal cord	Maximum dose (Gy)	36.92 ± 6.16	39.19 ± 5.07	0.193

*FA* + *C* full arcs plans with constraints, *OPA* + *C* optimal partial arcs plans with constraints, *PTV* planning target volume, *CI* conformity index, *HI* heterogeneity index.

*Represents significant difference (p < 0.05).

The whole lung V_5_, V_10_, V_15_, V_20_, and the MLD in OPA-C plans were significantly lower than FA-C plans (*p* < 0.05). On the contrary, the heart V_30_, V_40_, and MHD in FA-C plans were significantly lower than in OPA-C plans (*p* < 0.05) (Supplementary Table S1).

### The MUs, delivery time, and gamma passing rates

The FA + C plans required more MUs than OPA + C plans (673 vs. 605 MUs, *p* = 0.075) (Table 3). The delivery time was significantly longer in FA + C than that in OPA + C plans (237 vs. 192 s, *p* < 0.05) (Table 3). The mean gamma passing rates with 3%/3 mm of the FA + C and OPA + C plans were 97.67% ± 1.09% and 96.17% ± 0.75%. There were no significant differences between FA + C and OPA + C plans in gamma passing rates (Table 3). The FA + C and OPA + C plans passed the gamma criteria. The OPA angle using VBA in VMAT could effectively reduce the delivery time for EC, but it did not affect the MUs and gamma passing rates.

**Table 3:** The MUs and delivery time in FA + C and OPA + C plans.

Parameter	FA + C	OPA + C	*p*-value
MUs	673 ± 183	605 ± 139	0.075
Delivery time (s)	237 ± 8	192 ± 37	0.000*
Gamma passing rate (%)	97.67 ± 1.09	96.17 ± 0.75	0.139

*FA* + *C* full arcs plans with constraints, *OPA* + *C* optimal arcs plans with constraints, *MU* monitor unit, *s* second.

*Represents significant difference (p < 0.05).

The FA-C required fewer MUs than OPA-C plans (450 vs. 497 MUs, *p* = 0.001) (Supplementary Table S2). The delivery time was significantly longer in FA-C than in OPA-C plans (231 vs. 192 s, *p* < 0.05) (Supplementary Table S2).

## Discussion

Our study implemented the OPA angle based on the VBA in VMAT for clinical EC patients. The results showed that OPA + C plans could significantly reduce lung V_5_ and V_10_ compared with FA + C plans. Moreover, the doses to other normal tissues could also achieve the dose constraints. Therefore, this study indicated that VBA could provide the personalized OPA angle, which could be applied to clinical EC patients to improve treatment plans.

In the recent years, VMAT has been shown to be dosimetrically superior to IMRT^10, 24–26^. Gao et al.^27^ reported that compared with 7-field IMRT, VMAT showed better conformality and uniformity of the target. The whole lung V_5_ and V_20_ were 47% and 20% in VMAT, respectively. Zhang et al.^28^ compared VMAT with conventional sliding window IMRT to treat upper thoracic EC. The VMAT could effectively protect the lungs from dose irradiation and also reduce the number of MUs and treatment time. The average of the whole lung V_5_, V_10_, and V_20_ were 48%, 41%, and 19% in VMAT, respectively. Chen et al.^10^ indicated that compared to IMRT, VMAT could improve the target dose coverage and decrease the maximum dose of the spinal cord, MUs, and treatment time. VMAT could significantly decrease lung V_20_, V_25_, V_30_, V_35_, V_40_. For lung V_5_, VMAT was similar to IMRT. Lin et al.^29^ assessed VMAT for EC at all locations. They found that the patients with upper, middle, and lower esophageal tumors were 48%, 47%, 45% in whole lung V_5_ and 20%, 16%, 12% in whole lung V_20_. The major factors affecting lung V_5_ were the arc angle factor and the dose constraint factor. In our study, the purpose of comparing FA-C and OPA-C plans was to investigate the arc angle factor's effect and add the dose constraint factor to achieve the actual clinical situation. The OPA-C plans were not affected by the dose constraint factor and only the precise and personalized arc angle factor reducing the lung dose. Only one plan of lung V_5_ was less than 55% in the 30 FA-C plans. The average of the lung V_5_ was as high as 88% in FA-C plans. Sixteen plans of lung V_5_ were less than 55% in the 30 OPA-C plans. The average of the lung V_5_ decreased to 57%. After optimization with dose constraints, the whole lung V_5_ and V_10_ were 48% and 30% in FA + C plans, respectively, similar to previous studies. In the OPA + C plans, the whole lung V_5_ and V_10_ could achieve 43% and 28%, lower than the FA + C plans. The whole lung V_5_ and V_10_ could be decreased by using the OPA angles.

In this study, the personalized arc angle was calculated using VBA for lung doses in clinical patients with EC. Although each patient with EC could achieve the defined dose target with a full arc treatment plan, it required a lung dose constraint to do so. However, in the present study, by defining the expected lung V_5_, a corresponding arc angle, called the OPA angle, could be calculated in the VBA. The OPA angle was implemented in the treatment plan of EC patients to achieve the goal without dose constraints for the lung dose. This study also showed that the range of OPA angles was between 80° and 310° for 30 patients, with a very wide variation in the range of OPA angles used for each patient. According to the formula of the VBA, the width of the PTV, the length of the PTV, and the whole lung volume would affect the calculation of the OPA angle. Additionally, a personalized OPA angle effectively reduces the lung dose and helps the medical physicist quickly to set up the arc angle for each patient. Therefore, by comparing the difference between FA and OPA plans, it was found that a personalized OPA angle is necessary.

Gagliardi et al.^30^ indicated that when the heart V_30_ is higher than 45% or the MHD is higher than 26 Gy, the risk of pericarditis would increase. Wei et al.^31^ demonstrated that the risk of pericarditis was 73% and 13%, with heart V30 > 46% and V30 < 46%, respectively. In this study, the results demonstrated that the MHD and heart V30 were 20.76 Gy and 29% in the FA + C plans, and the MHD and heart V30 were 21.37 Gy and 30% in the OPA + C plans. Lin et al.^29^ assessed VMAT for EC at all locations. They found that the patients with upper, middle, and lower esophageal tumors were 40.48 Gy, 41.40 Gy, and 36.12 Gy in maximum spinal cord dose. Chen et al.^10^ found that the maximum spinal cord dose was 38.20 Gy for 391 EC patients. Our results showed that the maximum spinal cord dose was 36.92 Gy and 39.19 Gy in FA + C and OPA + C plans, similar to other studies mentioned above. There were no significant differences in heart and spinal cord doses between FA + C and OPA + C plans, which were similar to other studies mentioned above. This study indicated that the OPA angle applied to EC patients could significantly decrease lung V_5_ and V_10_ with acceptable doses to the heart and spinal cord in the RTP.

In tomotherapy, several studies reported reducing the lung dose by restricting the irradiated angle. Chang et al.^32^ said a substantial reduction in the lung dose using a fan-shaped complete block compared to a non-block design for middle thoracic EC in tomotherapy. Ito et al.^9^ indicated that a directional block with an angle of 50 or 60 degrees could reduce the lung dose for cervical EC in tomotherapy. In our previous study using VBA^15^, the angle of the complete block was equal to the restricted angle, which would be set to 360°-OPA angle to control the radiation angle in tomotherapy. Therefore, the OPA angle could also be used in tomotherapy for EC patients and restrict irradiated angle to control the lung V_5_ and V_10_.

Reducing delivery time could be beneficial for patients and institutions. Several studies investigated the delivery time in VMAT^14, 33–35^. Chen et al.^10^ found that the shorter delivery time of the VMAT technique may reduce patient discomfort during long-term treatment and improve delivery quality. Wala et al.^36^ indicated that using the optimal partial-arcs could minimize the delivery time without significantly affecting dose quality in VMAT. Jiang et al.^14^ showed the single/partial-arc VMAT (636 ± 108 and 384 ± 90 s, respectively) plan significantly reduced the treatment time compared to the IMRT (822 ± 156 s) plan. Especially, the partial-arc VMAT was the best to shorten the delivery time. In our study, the delivery time in OPA + C plans (192 ± 37 s) was shorter than in FA + C plans (237 ± 8 s). Therefore, the OPA angle used in VMAT plans could effectively reduce the delivery time to lead to better treatment quality. Additionally, the IMRT and VMAT are covered in our national healthcare insurance. VMAT not only produces a similar or better dose distribution than IMRT but also achieves a reduction in treatment time. Therefore, our institution usually uses the VMAT plans to treat esophageal cancer patients.

In our study, the prescribed dose of 45 Gy in 25 fractions was defined for esophageal cancer. Yang et al.^37^ compared the patients who received the lower dose (≤ 45 Gy) radiotherapy, and higher dose (> 45 Gy) radiotherapy for esophageal squamous cell carcinoma. They found that the higher dose radiotherapy does not increase pathological remission rate or improve overall survival, compared to lower dose radiotherapy. The lower radiation dose, including 40 Gy in 20 fractions, 41.4 Gy in 23 fractions, or 45 Gy in 25 fractions, might be a preferable time-dose fraction scheme. Therefore, we designed the prescribed dose of 45 Gy to simulate the esophageal cancer plans in Table 2. The results of 10 FA + C and 10 OPA + C plans in the prescribed dose of 50.4 Gy are shown in Supplementary Table S3.

The limitation of this study was that the skills and experience of operators might affect the quality of the plan. Even the VBA could provide the OPA angle to reduce the lung dose effectively, the doses constraints of OARs were still manually adjusted by operators during the optimization in RTP. Further prospective clinical studies enrolled more patients and operators are needed to verify VBA in RTP for EC.

## Conclusion

This study successfully applied VBA to RTP of clinical EC patients. The VBA could simplify the arc angle selection in RTP, provide a personalized OPA angle for each patient. The lung V_5_, V_10_, and delivery time could be significantly reduced while the lung V_20_ could be insignificantly reduced by using OPA angle in VMAT for EC.

## Supplementary Information

Supplementary Tables.
