# Supplementary material for: Planning evaluation of a novel volume-based algorithm for personalized optimization of lung dose in VMAT for esophageal cancer
Source: Sci Rep. 2022 Feb 15;12:2513. doi: 10.1038/s41598-021-04571-3 (PMC8847643; doi:10.1038/s41598-021-04571-3)
Supplement: Supplementary file 1 — Supplementary Tables. [file 41598_2021_4571_MOESM1_ESM.docx]

**Supplementary information**

**Planning evaluation of a novel volume-based algorithm for personalized optimization of lung dose in VAMT for esophageal cancer**

Authors：

Chen-Xiong Hsu^1,2^, Kuan-Heng Lin^1,2,3^, Shan-Ying Wang^1,4^, Wei-Ta Tsai^1^, Chiu-Han Chang^2^, Hui-Ju Tien^1,2^, Pei-Wei Shueng^2,5*^, Tung-Hsin Wu^1*^, Greta S. P. Mok^6^

Affiliations：

^1^Department of Biomedical Imaging and Radiological Sciences, National Yang Ming Chiao Tung University, Taipei, Taiwan

^2^Division of Radiation Oncology, Far Eastern Memorial Hospital, New Taipei City, Taiwan

^3^Industrial Ph.D. Program of Biomedical Science and Engineering, National Yang Ming Chiao Tung University, Taipei, Taiwan

^4^Department of Nuclear Medicine, Far Eastern Memorial Hospital, New Taipei City, Taiwan

^5^ Faculty of Medicine, School of Medicine, National Yang Ming Chiao Tung University, Taipei, Taiwan

^6^ Biomedical Imaging Laboratory, Department of Electrical and Computer Engineering, Faculty of Science and Technology, University of Macau, Macau, SAR, China.

These authors contributed equally: Chen-Xiong Hsu and Kuan-Heng Lin

Correspondence: Pei-Wei Shueng (email: shuengsir@gmail.com) and Tung-Hsin Wu (email: tung@ym.edu.tw) ,Tel：+8862-2826-7061, Fax： +8862-2820-1095

Table S1. Comparison of dosimetric factors between FA-C and OPA-C plans.

| Parameter | |  | FA-C | OPA-C | *p*-value |  |
| --- | --- | --- | --- | --- | --- | --- |
| PTV |  |  |  |  |  |  |
|  | D_5_ (Gy) |  | 46.36 ± 7.99 | 47.33 ± 8.42 | 0.167 |  |
|  | D_95_ (Gy) |  | 43.80 ± 7.66 | 43.70 ± 7.49 | 0.796 |  |
|  | HI |  | 1.06 ± 0.02 | 1.08 ± 0.03 | 0.004* |  |
|  | CI |  | 1.31 ± 0.30 | 2.22 ± 0.72 | 0.000* |  |
| Whole lung |  |  |  |  |  |  |
|  | Mean dose (Gy) |  | 17.22 ± 3.58 | 13.62 ± 2.86 | 0.000* |  |
|  | V_20_ (%) |  | 32.25 ± 11.61 | 25.95 ± 7.64 | 0.017* |  |
|  | V_15_ (%) |  | 50.71 ± 14.45 | 36.13 ± 9.29 | 0.000* |  |
|  | V_10_ (%) |  | 73.04 ± 16.53 | 44.18 ± 9.35 | 0.000* |  |
|  | V_5_ (%) |  | 88.21 ± 15.10 | 57.04 ± 9.44 | 0.000* |  |
| Right lung |  |  |  |  |  |  |
|  | Mean dose (Gy) |  | 16.18 ± 3.54 | 12.49 ± 3.06 | 0.000* |  |
|  | V_20_ (%) |  | 27.86 ± 10.96 | 22.29 ± 7.64 | 0.027* |  |
|  | V_15_ (%) |  | 44.14 ± 13.66 | 30.04 ± 7.99 | 0.000* |  |
|  | V_10_ (%) |  | 66.29 ± 16.79 | 39.75 ± 8.84 | 0.000* |  |
|  | V_5_ (%) |  | 88.07 ± 15.15 | 53.89 ± 10.79 | 0.000* |  |
| Left lung |  |  |  |  |  |  |
|  | Mean dose (Gy) |  | 18.45 ± 4.12 | 14.45 ± 3.45 | 0.000* |  |
|  | V_20_ (%) |  | 37.75 ± 14.28 | 28.14 ± 9.73 | 0.001* |  |
|  | V_15_ (%) |  | 58.68 ± 17.93 | 36.29 ± 9.87 | 0.000* |  |
|  | V_10_ (%) |  | 79.39 ± 19.22 | 46.11 ± 10.29 | 0.000* |  |
|  | V_5_ (%) |  | 89.21 ± 15.97 | 60.18 ± 10.77 | 0.000* |  |
| Heart |  |  |  |  |  |  |
|  | Mean dose (Gy) |  | 20.57 ± 8.44 | 26.70 ± 10.58 | 0.003* |  |
|  | V_40_ (%) |  | 11.68 ± 9.56 | 27.18 ± 20.10 | 0.002* |  |
|  | V_30_ (%) |  | 24.14 ± 16.38 | 46.89 ± 26.86 | 0.001* |  |
| Spinal cord |  |  |  |  |  |  |
|  | Maximum dose (Gy) |  | 44.74 ± 8.81 | 48.65 ± 8.34 | 0.030* |  |
| *Abbreviations: FA-C = full arcs plans without constraints; OPA-C = optimal arcs plans without constraints; PTV = planning target volume; CI = conformity index; HI = heterogeneity index*  ** represents significant difference (p < 0.05)* | | | | | | |

Table S2. The MU and delivery time in FA-C and OPA-C plans.

| Parameter |  | FA-C | OPA-C | *p*-value |
| --- | --- | --- | --- | --- |
| MUs |  | 450 ± 114 | 497 ± 67 | 0.001* |
| Delivery time (s) |  | 231 ± 19 | 192 ± 38 | 0.000* |
| *Abbreviations:  FA-C = full arcs plans without constraints; OPA-C = optimal arcs plans without constraints; MU = monitor unit; s = second*  ** represents significant difference (p < 0.05)* | | | | |

Table S3. Comparison of dosimetric factors between FA+C and OPA+C plans in the prescribed dose of 50.4 Gy for 10 esophageal cancer patients.

| Parameter | |  | | FA+C | OPA+C | *p*-value |
| --- | --- | --- | --- | --- | --- | --- |
| PTV |  |  |  |  |  |  |
|  | HI |  |  | 1.08 ± 0.03 | 1.07 ± 0.03 | 0.601 |
|  | CI |  |  | 1.09 ± 0.08 | 1.08 ± 0.06 | 0.741 |
| Whole lung |  |  |  |  |  |  |
|  | Mean dose (Gy) |  |  | 10.27 ± 1.03 | 9.64 ± 1.53 | 0.298 |
|  | V_20_ (%) |  |  | 17.10 ± 2.73 | 16.50 ± 3.98 | 0.699 |
|  | V_15_ (%) |  |  | 21.50 ± 3.14 | 20.10 ± 4.18 | 0.408 |
|  | V_10_ (%) |  |  | 28.30 ± 3.97 | 25.50 ± 4.35 | 0.150 |
|  | V_5_ (%) |  |  | 45.20 ± 5.53 | 39.00 ± 5.64 | 0.023* |
| Heart |  |  |  |  |  |  |
|  | Mean dose (Gy) |  |  | 19.74 ± 8.30 | 19.80 ± 8.67 | 0.988 |
|  | V_40_ (%) |  |  | 17.80 ± 11.37 | 18.40 ± 11.98 | 0.910 |
|  | V_30_ (%) |  |  | 30.70 ± 16.43 | 30.40 ± 17.00 | 0.968 |
| Spinal cord |  |  |  |  |  |  |
|  | Maximum dose (Gy) |  |  | 43.69 ± 1.51 | 43.76 ± 1.33 | 0.909 |
| *Abbreviations:  FA+C = full arcs plans with constraints; OPA+C = optimal arcs plans with constraints; PTV = planning target volume; CI = conformity index; HI = heterogeneity index*  ** represents significant difference (p < 0.05)* | | | | | | |
